# The Development of Mouse APECED Models Provides New Insight into the Role of AIRE in Immune Regulation

**DOI:** 10.1080/17402520500212589

**Published:** 2005-09

**Authors:** Lara E. Pereira, Pavel Bostik, Aftab A. Ansari

**Affiliations:** Department of Pathology and Laboratory Medicine Emory University Atlanta GA USA

## Abstract

Autoimmune polyendocrinopathy candidiasis ectodermal dystrophy is a rare recessive autoimmune disorder caused by a defect in a single gene called AIRE (autoimmune regulator). Characteristics of this disease include a variable combination of autoimmune endocrine tissue destruction, mucocutaneous candidiasis and ectodermal dystrophies. The development of Aire-knockout mice has provided an invaluable model for the study of this disease. The aim of this review is to briefly highlight the strides made in APECED research using these transgenic murine models, with a focus on known roles of Aire in autoimmunity. The findings thus far are compelling and prompt additional areas of study which are discussed.


*Clinical & Developmental Immunology*, September 2005; 12(3): 211–216

Taylor & Francis

Taylor & Francis Group

### The development of mouse APECED models provides new insight into the role of AIRE in immune regulation

LARA E. PEREIRA, PAVEL BOSTIK, & AFTAB A. ANSARI

*Department of Pathology and Laboratory Medicine, Emory University, Atlanta, GA, USA*

**Abstract**
Autoimmune polyendocrinopathy candidiasis ectodermal dystrophy is a rare recessive autoimmune disorder caused by a defect in a single gene called AIRE (autoimmune regulator). Characteristics of this disease include a variable combination of autoimmune endocrine tissue destruction, mucocutaneous candidiasis and ectodermal dystrophies. The development of Aire-knockout mice has provided an invaluable model for the study of this disease. The aim of this review is to briefly highlight the strides made in APECED research using these transgenic murine models, with a focus on known roles of Aire in autoimmunity. The findings thus far are compelling and prompt additional areas of study which are discussed.

**Keywords:** *APECED, AIRE, autoimmunity, immune regulation, thymocyte deletion*

### Introduction

Autoimmune polyendocrinopathy candidiasis ectodermal dystrophy (APECED), also known as autoimmune polyglandular syndrome Type I (APS I), is a rare autosomal recessive disorder that is characterized by autoimmunity against organ-specific autoantigens (Ahonen 1985, Straub and Manns 1998, Aaltonen and Bjorses 1999). The disease affects primarily endocrine organs causing a number of conditions that include adrenocortical and ovarian failure, insulin-dependent diabetes, hypoparathyroidism and hepatitis (Ahonen 1985, Clemente et al. 1997, Straub and Manns 1998, Aaltonen and Bjorses 1999). This syndrome is also characterized by immunodeficiency as exemplified by enhanced susceptibility of patients to infection and development of chronic mucocutaneous candidiasis (Ahonen 1985, Straub and Manns 1998, Aaltonen and Bjorses 1999). The disorder is prevalent among Finns, Sardinians, and Iranian Jews and usually manifests during childhood with additional pathological conditions emerging later in life (Shapiro et al. 1987, Straub and Manns 1998, Bjorses et al. 1996, Rosatelli et al. 1998). APECED is treated by tackling the specific problems of the disease, such as replacing hormones that are in short supply, providing immunosuppressive therapy and treating the *Candida albicans* infection (Ahonen et al. 1986, Faulds et al. 1993, Seidman et al. 1990). There is no known cure at present for APECED.

Usually autoimmune diseases involve defects in several genes, such as those that encode T-cell receptors, immunoglobulins and cytokines. APECED is unique in that it is caused by a mutation in a single gene. A positional cloning approach aided the identification of the autoimmune regulator gene AIRE (Nagamine et al. 1997). This gene, which is localized to chromosome 21 in humans, is split into 14 exons over ~13 kb (Aaltonen et al. 1994, The Finnish-German APECED Consortium 1997, Nagamine et al. 1997). A number of APECED mutations have been identified so far and these mutations typically include either single nucleotide substitutions or small insertions/deletions in AIRE (Pearce et al. 1998, Wang et al. 1998, Heino et al. 2001, Podkrajsek et al. 2005). The mutations usually introduce a premature termination codon, resulting in a truncated protein, or disrupt one or more of the functional domains of AIRE (Heino et al. 2001,

Correspondence: A. A. Ansari, Department of Pathology and Laboratory Medicine, Emory University, 101 Woodruff Circle, Room # 2309, Atlanta, GA 30329, Tel: 404 712 2834, Fax : 404 712 1771. E-mail: pathaaa@emory.edu

ISSN 1740-2522 print/ISSN 1740-2530 online © 2005 Taylor & Francis
DOI: 10.1080/17402520500212589

Halonen et al. 2004, Meloni et al. 2005). The most common mutation is a C to T change at nucleotide 889 in exon 6, which results in a premature stop codon (Wang et al. 1998). A 13 bp deletion in exon 8 (bases 1094–1106) appears to be the second most common mutation (Pearce et al. 1998, Wang et al. 1998).

### The characterization of AIRE

Several genetic and molecular approaches have shed light on the biochemical nature of AIRE, providing some insight into the function of this protein. AIRE encodes a ~57 kDa protein composed of ~552 amino acids (Aaltonen et al. 1994, Nagamine et al. 1997, Aaltonen and Bjorses 1999). The protein possesses a nuclear localization domain, an HSR (homogeneously staining region) homomultimerization domain, a SAND (*S*p100, *A*IRE-1, *N*ucP41/75, and *D*EAF-1/suppressin) domain, 2 plant-homeodomain (PHD)-type cysteine-rich zinc-finger domains and four LXXLL motifs, suggesting a role for this protein as a transcriptional regulator (Aaltonen and Bjorses 1999, Gibson et al. 1998, The Finnish-German APECED Consortium 1997). Indeed, experiments have demonstrated that upon phosphorylation and dimerization, AIRE binds to DNA sequences that are known to be recognized by other proteins that, like AIRE, possess zinc-finger and leucine-zipper motifs (Kumar et al. 2001, Purohit et al. 2005). *In vitro* experiments including gel-shift assays revealed that AIRE has a high binding affinity for two sequences—a TATA-like box (TTATTA) and a tandem repeat of a G-box (ATTGGTTA) (Kumar et al. 2001, Purohit et al. 2005). The activation of reporter genes, such as luciferase and chloramphenicol transacetylase, by AIRE fused to the GAL4 DNA-binding domain suggests a transcriptional activation function for AIRE (Bjorses et al. 2000, Pitkänen et al. 2000). A study has shown that AIRE also possesses E3 ligase activity, which facilitates the polyubiquitination of substrates (Uchida et al. 2004). It has been suggested that the E3 ligase activity is required for the modification of AIRE-interacting proteins so that AIRE can localize correctly to the site of transcriptional activation (Uchida et al. 2004). Studies have shown that the systematic deletion or modification of one or more of the functional domains of AIRE interferes with the stability, transcriptional activation properties or nuclear targeting of the protein, highlighting the importance of each domain (Rinderle et al. 1999, Bjorses et al. 2000, Ramsey et al. 2002a, Halonen et al. 2004, Meloni et al. 2005).

### The mouse APECED model: Elucidating the role of Aire

A mouse homolog (Aire) of the human AIRE gene was identified and a comparison between the two has revealed a ~77% nucleotide homology in coding regions and ~71% protein homology (Antonarakis et al. 1998, Halonen et al. 1998, Yaspo et al. 1998, Wang et al. 1999, Blechschmidt et al. 1999). This prompted the successful development of a mouse model for APECED to facilitate in-depth studies of this disease. Aire-deficient mice exhibit characteristics similar to APECED patients, which include auto-reacting antibodies against certain organ-specific antigens and multi-organ lymphocyte infiltration (Aaltonen and Bjorses 1999, Ramsey et al. 2002b, Kuroda et al. 2005). As with humans, the endocrine organs are the main targets of the autoimmune response in mice resulting in similar phenotypes (Ramsey et al. 2002b). The mouse model has therefore enabled further study of the role of Aire in autoimmunity and studies thus far suggest that the autoimmune response is likely a result of the dysregulation in thymocyte clonal deletion of T-cells within the thymus (negative selection), which is regulated by Aire.

This view was formally established by the use of double transgenic mice which were bred to include a TCR transgene and an Aire null mutation. A failure to delete the TCR transgene-expressing T-cells demonstrated the link between Aire and the elimination of autoreactive T-cells (Zuklys et al. 2000, Anderson et al. 2002, Liston et al. 2003, Park et al. 2003). One such study traced the fate of hen egg lyzozyme (HEL)-specific T-cells, expressed in mice, in the presence or absence of Aire (Liston et al. 2003). An almost complete failure to delete autoreactive HEL-specific T-cells was observed in knockout mice thymi, suggesting that Aire regulates the negative selection of self-reactive T-cells in the thymus (Liston et al. 2003). It has been proposed that such negative selection is facilitated by promoting the transcription of a number of self-antigens in thymic medullary epithieal cells (MECs). These antigens are presented to developing T-cells and those that are self-reactive are deleted. The transcriptional control of these peripheral self-antigens by Aire was hypothesized because both Aire and the self-antigens are expressed in MECs (Heino et al. 1999, Zuklys et al. 2000, Derbinski et al. 2001, Halonen et al. 2001, Anderson et al. 2002, Adamson et al. 2004). Indeed, a comparison of gene profiles from Aire-knockout mice with wild-type littermates revealed a lowered gene expression coding for several peripheral antigens in MECs from the knockouts, suggesting the requirement of Aire for the expression of the self-antigen (Anderson et al. 2002). However, autoimmunity against the ubiquitous protein α-fodrin was shown to occur in Aire-negative mice but the expression of this protein was retained in the thymi of these knockouts (Kuroda et al. 2005). This suggests that the role of Aire in clonal deletion is not limited to the transcriptional control of self-antigens in the thymus.

Aire may also regulate the modification and/or presentation of self-antigens so that they are efficiently recognized by autoreactive T-cells.

Quantification of $CD4^{+}CD25^{+hi}$ regulatory T-cells (Tregs) in the TCR-transgenic systems described above revealed no significant difference in cell number in the Aire-deficient and wild-type backgrounds (Liston et al. 2003). *In vitro* and *in vivo* approaches also demonstrated that the normal suppressive function of these cells was retained (Liston et al. 2003). Thus, a deficiency in regulatory T-cells or a defect in their suppressive function does not appear to be the cause of the central tolerance defect in Aire-knockout mice.

Aire also appears to exert a quantitative genetic effect on thymic clonal deletion (Liston et al. 2004). Levels of HEL mRNA were measured in different Aire transgenic mice. Thymic HEL mRNA was absent in $Aire^{-/-}$ mice and levels of this transcript were also reduced to an intermediate level in $Aire^{+/-}$ heterozygotes when compared to wild-type mice. In agreement with this data, heterozygosity also had an impact on the efficiency of thymic clonal deletion. HEL-specific T-cells were tracked in the thymi of these transgenic mice and a significant defect in clonal deletion was observed in $Aire^{-/-}$ mice while an intermediate defect was seen in $Aire^{+/-}$ mice. Circulating reactive HEL T-cells were higher in number in Aire heterozygotes and still higher in Aire null knockouts when compared to wild-type mice. This study is in agreement with the observation made by Sediva and colleagues, whose research showed an occurrence of a subclinical immune deficit in humans heterozygous for the AIRE mutation (Sediva et al. 2002). Carriers exhibited higher levels of certain immunoglobulins (IgA) and activated T-cells when compared to non-carriers.

In addition to resolving the role of Aire in autoimmunity, a study by Chin et al focused on factors that influence the induction and regulation of Aire itself. Experimental data suggests that the expression of Aire is controlled by lymphotoxin which also mediates lymph node organogenesis and inflammatory responses (Chin et al. 2003). As with Aire, lymphotoxin is also expressed in the thymi. Lymphotoxin α and lymphotoxin β-receptor knockout mice possess thymi that produce lower levels of Aire mRNA. However, it is unclear if these results are a direct effect of lymphotoxin on Aire transcription or due to a defect in thymi development in these knockouts.

### Conclusions and future directions

Although significant advances have been made in our understanding of the role of Aire in organ-specific tolerance, little is known about other functions of this protein. For instance, the immunodeficiency in APECED cannot be explained simply by the failure of clonal deletion. The reason for Aire production in other organs and cells, such as the pancreas and macrophages respectively, is also unclear but clearly suggests additional roles of Aire. It would be interesting to explore the effect of external factors, such as infection, on the pathogenesis of this disease. These conditions would more aptly reflect scenarios experienced by APECED patients.

There is some experimental data that addresses the role of Aire in other areas of immune regulation. A study by Sato and colleagues showed that Aire downregulates IL-1 receptor antagonist (IL-1Ra) and this decrease is due to the competition of Aire for the transcriptional co-activator CREB-binding protein (CBP) (Sato et al. 2004). The sequestration of CBP by Aire results in decreased expression of IL-1Ra which usually inhibits the biological activity of IL1 that is involved in the defense against *Candida albicans*. Thus, in the absence of Aire, IL-1Ra is upregulated, inhibiting the function of IL-1 and this is likely to contribute to the immunodeficiency observed in APECED patients. The same study showed that Aire-expressing cells exhibit a downregulation of major histocompatibility complex II molecules, which is important for acquired immunity. This study highlights that the function of Aire is not limited to transcriptional activation alone but also involves repression. Indeed, a recent study using a microarray approach showed that a number of genes are repressed by Aire and a subset of these genes encode molecules other than self-antigens, such as those involved in antigen presentation (Johnnidis et al. 2005). It would be interesting to explore further if Aire influences the levels and/or functions of additional immune response cell lineages such as those involved in innate immunity. Other studies could also pursue the influence of Aire on the polarity of the immune system—this would involve investigating how an Aire mutation affects the balance between cytokine profile-based type 1 and type 2 immune responses which eventually determine if the immune system elicits a cell-mediated or humoral response. Another avenue of research that would be of interest to pursue is the role, if any, of Aire in other autoimmune diseases that are for instance, based on molecular mimicry—is the expression or function of Aire affected in such diseases and if so, how does this contribute to the autoimmune response.

Although current Aire-knockout systems have greatly facilitated the study of APECED and the role of Aire, our understanding is still limited in that the detailed molecular basis for the changes that occur in the immune system following an Aire mutation are unclear. As of now, we have a great deal of insight on the effect of Aire-deficiency on the immune system since knockouts are born with the defect. If it were possible to temporally control an Aire mutation during

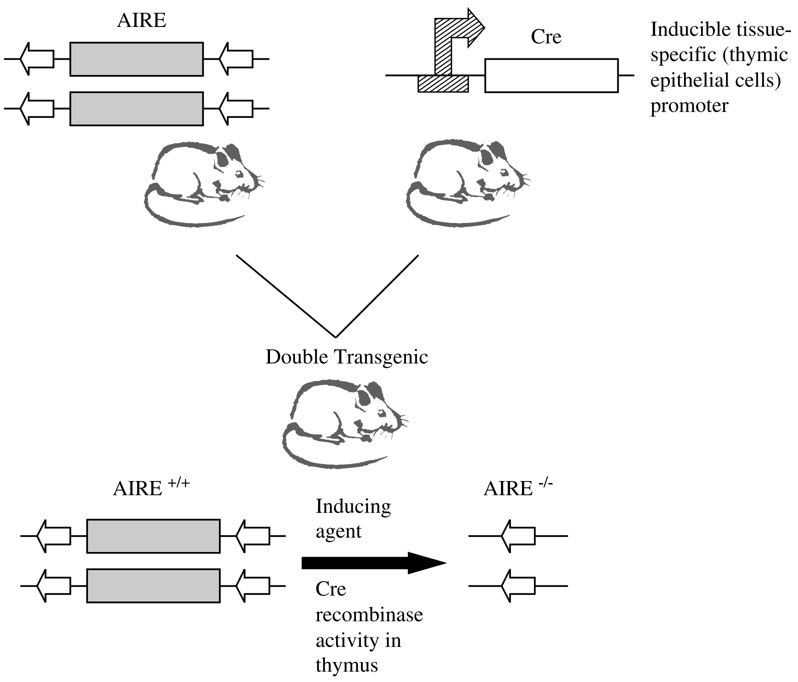

Figure 1. Strategy to temporally regulate AIRE deletion using transgenic mice. Two mice lines are generated; one possessing the *loxP*-flanked (indicated by open arrows) AIRE, and the other has Cre under the control of an inducible promoter that is active only in specific tissues. Mating of the two transgenic mice will result in a double-transgenic mouse in which *loxP*-flanked AIRE is deleted in a time-specific manner by Cre activity but only upon introduction of a specific inducer into the mouse. Ideally, this deletion should occur in thymic epithileal cells where AIRE is primarily expressed.

the life cycle of a model organism such as mice, this would open the door to several avenues of research. Creating a conditional Aire knockout would permit such studies. The Cre/lox system has been widely used to alter target genes in mice (Lakso et al. 1992, Sauer 1998, Dragatsis and Zeitlin 2001). Typically, the target gene is flanked by LoxP (specific locus of crossing over) sites which are recognized by the Cre recombinase. This enzyme, which is originally from bacteriophage P1, cleaves DNA at the LoxP site and catalyzes a recombination reaction effectively deleting the gene flanked by the two LoxP sites. The strategy to create a conditional Aire knockout involves generating two independent transgenic mouse lines: one would have an inducible promoter that drives the expression of Cre-recombinase in a specific cell lineage (for example, thymus epithelial cells) that produce Aire, and the other would be homozygous for the target Aire gene with LoxP sequences introduced at specific sites within, or flanking Aire (Figure 1). The progeny of these two murine lines would allow for Cre-mediated deletion of Aire, effectively creating an Aire knockout but only when induced by a specific agent. By controlling the age at which the gene can be deleted, one can monitor what changes occur in the immune system once the defect is introduced. Other questions that could be addressed include how abrupt is the change, how soon do symptoms manifest, and how age influences the manifestation of the disease in mice.

In conclusion, APECED is the only known autoimmune disease in humans that is caused by a mutation in a single gene. Aire knockout mice exhibit many symptoms similar to APECED patients and this has facilitated the study of this disorder in great detail, leading to an improved understanding of the workings of the immune system and the autoimmune response. Elucidating the role of Aire in both autoimmunity and immunodeficiency using murine models is reasoned to further our understanding of the immune system and will facilitate the development of future therapeutics.

### References

Aaltonen J, Bjorses P. 1999. Cloning of the APECED gene provides new insight into human autoimmunity. Ann Med 31:111–116.

Aaltonen J, Bjorses P, Sandkuijl L, Perheentupa J, Peltonen L. 1994. An autosomal locus causing autoimmune disease: Autoimmune polyglandulardisease type I assigned to chromosome 21. Nat Genet 8:83–87.

Adamson KA, Pearce SH, Lamb JR, Seckl JR, Howie SE. 2004. A comparative study of mRNA and protein expression of the autoimmune regulator gene (Aire) in embryonic and adult murine tissues. J Pathol 202:180–187.

Ahonen P. 1985. Autoimmune polyendocrinopathy—candidosis—ectodermaldystrophy (APECED): Autosomal recessive inheritance. Clin Genet 27:535–542.

Ahonen P, Myllarniemi S, Kahanpaa A, Perheentupa J. 1986. Ketoconazole is effective against chronic mucocutaneous candidiasis of autoimmune polyendocrinopathy-candidosis-ectodermal dystrophy (APECED). Acta Med Scand 220:333–339.

Anderson MS, Venanzi ES, Klein L, Chen Z, Berzins SP, Turley SJ, Bohmer HV, Bronson R, Dierich A, Benoist C, Mathis D. 2002. Projection of an immunological self shadow within the thymus by the Aire protein. Science 298:1395–1401.

Antonarakis SE, Mittaz L, Heino M, Peterson P, Chen Q, Maclaren NK. 1998. Mutation analyses of AIRE in autoimmune polyglandular syndrome type 1, immunohistochemical studies and isolation of the mouse gene. Am J Hum Genet 63:A349.

Bjorses P, Aaltonen J, Vikman A, Perheentupa J, Ben-Zion G, Chiumello G, Dahl N, Heideman P, Hoorweg-Nijman JJ, Mathivon L, Mullis PE, Pohl M, Ritzen M, Romeo G, Shaipro MS, Smth CS, Solyom J, Zlotogora J, Petlonen L. 1996. Genetic homogeneity of autoimmune polyglandular disease type I. Am J Hum Genet 59:879–886.

Bjorses P, Halonen M, Palvimo JJ, Kolmer M, Aaltonen J, Ellonen P, Perheentupa J, Ulmanen I, Peltonen L. 2000. Mutations in the *AIRE* gene: Effects on subcellular localization and transactivation function of the autoimmune polyendocrinopathy-candidiasis-ectodermal dystrophy protein. Am J Hum Genet 66:378–392.

Blechschmidt K, Schweiger M, Wertz K, Poulson R, Christensen H-M, Rosenthal A, Lehrach H, Yaspo M-L. 1999. The mouse Aire gene: Comparative genomic sequencing, gene organization, and expression. Genome Res 9:158–166.

Chin RK, Lo JC, Kim O, Blink SE, Christiansen PA, Peterson P, Wang Y, Ware C, Fu YX. 2003. Lymphotoxin pathway directs thymic Aire expression. Nat Immunol 4:1121–1127.

Clemente MG, Obermayer-Straub P, Meloni A, Strassburg CP, Arangino V, Tukey RH, Virgiliis Sd, Manns MP. 1997. Cytochrome P450 1A2 is a hepatic autoantigen in autoimmune polyglandular syndrome type 1. J Clin Endocrinol Metab 82:1353–1361.

Derbinski J, Schulte A, Kyewski B, Klein L. 2001. Promiscuous gene expression in medullary thymic epithileal cells mirror the peripheral self. Nat Immunol 2:1032–1039.

Dragatsis J, Zeitlin S. 2001. A method for the generation of conditional gene repair mutations in mice. Nucleic Acids Res 29:e10.

Faulds D, Goa KL, Benfield P. 1993. Cyclosporin. A review of its pharmacodynamic and pharmacokinetic properties, and therapeutic use in immunoregulatory disorders. Drugs 45:953–1040.

Gibson TJ, Ramu C, Gemund C. 1998. The APECED polyglandular autoimmune syndrome protein, AIRE-1, contains the SAND domain and is probably a transcription factor. Trends Biochem Sci 23:242–244.

Halonen M, Bjorses P, Horelli-Kuitunen N, Palotie A, Jalanko A, Ulmanen I. 1998. The mouse homologue for the AIRE gene. Am J Hum Genet 63:A364.

Halonen M, Pelto-Huikko M, Eskelin P, Peltonen L, Ulmanen I, Kolmer M. 2001. Subcellular location and expression pattern of autoimmune regulator (*Aire*), the mouse orthologue for human gene defective in autoimmune polyendocrinopathy-candidiasis-ectodermal dystrophy (APECED). J Histochem Cytochem 49:197–208.

Halonen M, Kangas H, Ruppell T, Ilmarinen T, Ollila J, Kolmer M, Saarela JV, Ulmanen I, Eskelin P. 2004. APECED-causing mutations in AIRE reveal the functional domains of the protein. Hum Mutat 23:245–257.

Heino M, Peterson P, Kudoh J, Nagamine K, Lagerstedt A, Ovod V, Ranki A, Rantala I, Nieminen M, Tuukkanen J, Scott HS, Antonarakis SE, Shimizu N, Krohn K. 1999. Autoimmune regulator is expressed in the cells regulating immune tolerance in thymus medulla. Biochem Biophys Res Commun 257:821–825.

Heino M, Peterson P, Kudoh J, Shimizu N, Antonarakis SE, Scott HS, Krohn K. 2001. APECED mutations in the autoimmune regulator (AIRE) gene. Hum Mutat 18:205–211.

Johnnidis JB, Venanzi ES, Taxman DJ, Ting JP-Y, Benoist CO, Mathis DJ. 2005. Chromosomal clustering of genes controlled by the aire transcription factor. Proc Natl Acad Sci USA 102:7233–7238.

Kumar PG, Laloraya M, Wang CY, Ruan QG, Davoodi-Semiromi A, Kao KJ, She JX. 2001. The autoimmune regulator (AIRE) is a DNA-binding protein. J Biol Chem 276:41357–41364.

Kuroda N, Mitani T, Takeda N, Ishimaru N, Arakaki R, Hayashi Y, Bando Y, Izumi K, Takahashi T, Nomura T, Sakaguchi S, Ueno T, Takahama Y, Uchida D, Sun S, Kajiura F, Mouri Y, Han H, Matsushima A, Yamada G, Matsumoto M. 2005. Development of autoimmunity against transcriptionally unrepressed target antigen in the thymus of aire-deficient mice. J Immunol 174:1862–1870.

Lakso M, Sauer B, Mosinger Jr., B, Lee EJ, Manning RW, Yu SH, Mulder KL, Westphal H. 1992. Targeted oncogene activation by site-specific recombination in transgenic mice. Proc Natl Sci USA 89:6232–6236.

Liston A, Lesage S, Wilson J, Peltonen L, Goodnow C. 2003. Aire regulates negative selection of organ-specific T cells. Nat Immunol 4:350–354.

Liston A, Gray DHD, Lesage S, Fletcher AL, Wilson J, Webster KE, Scott HS, Boyd RL, Peltonen L, Goodnow CC. 2004. Gene dosage-limiting role of *Aire* in thymic expression, clonal deletion, and organ-specific autoimmunity. J Exp Med 200:1015–1026.

Meloni A, Fiorillo E, Corda D, Perniola R, Cao A, Rosatelli MC. 2005. Two novel mutations of the AIRE protein affecting its homodimerization properties. Hum Mutat 25:319.

Nagamine K, Peterson P, Scott H, Kudoh J, Minoshima S, Heino M, Krohn KJE, Lahoti MD, Mullis PE, Antonarakis SE, Kawasaki K, Asakawa S, Ito F, Shimizu N. 1997. Positional cloning of the APECED gene. Nat Genet 17:393–398.

Park Y, Moon Y, Chung HY. 2003. AIRE-1 (autoimmune regulator type 1) as a regulator of the thymic induction of negative selection. Ann N Y Acad Sci 1005:431–435.

Pearce SHS, Cheetham T, Imrie H, Vaidya B, Barnes ND, Bilous RW, Carr D, Meeran K, Shaw NJ, Smith CS, Toft AD, Williams G, Kendall-Taylor P. 1998. A common and recurrent 13-bp deletion in the autoimmune regulator gene in British kindreds with autoimmune polyendinocrinopathy type 1. Am J Hum Genet 63:1675–1684.

Pitkänen J, Doucas V, Sternsdorf T, Nakajima T, Aratani S, Jensen K, Will H, Vähämurto P, Ollila J, Vihinen M, Scott HS, Antonarakis SE, Kudoh J, Shimizu N, Krohn K, Peterson P. 2000. The autoimmune regulator protein has transcriptional transactivating properties and interacts with the common coactivator CREB-binding protein. J Biol Chem 275: 16802–16809.

Podkrajsek KT, Bratanic N, Krzisnik C, Battelino T. 2005. AIRE-1 mRNA analysis in a novel intronic mutation and two additional novel AIRE gene mutations in a cohort of APECED patients. J Clin Endocrinol Metab Epub.

Purohit S, Kumar PG, Laloraya M, She JX. 2005. Mapping DNA-binding domains of the autoimmune regulator protein. Biochem Biophys Res Commun 327:939–944.

Ramsey C, Bukrinsky A, Peltonen L. 2002a. Systematic mutagenesis of the functional domains of Aire reveals their role in intracellular targeting. Hum Mol Genet 11:3299–3308.

Ramsey C, Winqvist O, Puhakka L, Halonen M, Moro A, Kampe O, Eskelin P, Pelto-Huikko M, Peltonen L. 2002b. Aire deficient mice develop multiple features of APECED phenotype and show altered immune response. Hum Mol Genet 11:397–409.

Rinderle C, Christensen H-M, Schweiger S, Lehrach H, Laspo M-L. 1999. AIRE encodes a nuclear protein co-localizing with cytoskeletal filaments: Altered subcellular distribution of mutants lacking PHD-zinc fingers. Hum Mol Genet 8:277–290.

Rosatelli MC, Meloni A, Devoto M, Cao A, Scott HS, Peterson P, Heino M, Krohn KJ, Nagamine K, Kudoh J, Shimizu N, Antonarakis SE. 1998. A common mutation in Sardinian autoimmune polyendocrinopathy-candidiasis-ectodermal dystrophy patients. Hum Genet 103:428–434.

Sato K, Sato U, Tateishi S, Kubo K, Horikawa R, Mimura T, Yamamoto K, Kanda H. 2004. Aire downregulates multiple molecules that have contradicting immune-enhancing and immune-suppressive functions. Biochem Biophys Res Commun 318:935–940.

Sauer B. 1998. Inducible gene targeting in mice using the Cre/lox system. Methods 14.

Sediva A, Cihakova D, Lebl J. 2002. Immunological findings in patients with autoimmune polyendocrinopathy-candidiasis-ectodermal dystrophy (APECED) and their family members: Are heterozygotes subclinically affected? J Pediatr Endocrinol Metab 15:1491–1496.

Seidman E, Lacaille F, Russo P, Galeano N, Murphy G, Roy C. 1990. Successful treatment of autoimmune enteropathy with cyclosporine. J Ped 117:929–932.

Shapiro MS, Zamir R, Weiss E, Radnay J, Shenkman L. 1987. The polyglandular deficiency syndrome: a new variant in Persian Jews. J Endocrin Invest 10:1–7.

Straub PO, Manns MP. 1998. Autoimmune polyglandular syndromes. Balliere's Clinical Gastroenterol 12:293–315.

The Finnish-German APECED Consortium 1997. An autoimmune disease, APECED, caused by mutations in a novel gene featuring two PHD-type zinc-finger domains. Nat Genet 17:399–403.

Uchida D, Hatakeyama S, Matsushima A, H H, Ishido S, Hotta H, Kudoh J, Shimizu N, Doucas V, Nakayama KI, Kuroda N, Matsumoto M. 2004. AIRE functions as an E3 ubiquitin ligase. J Exp Med 199:167–172.

Wang CY, Davoodi-Semiromi A, Huang W, Connor E, Shi JD, She JX. 1998. Characterization of mutations in patients with autoimmune polyglandular syndrome type 1 (APS1). Hum Genet 103:681–685.

Wang CY, Shi JD, Davoodi-Semiromi A, Shi JX. 1999. Cloning of *Aire*, the mouse homologue of the autoimmune regulator (*AIRE*) gene responsible for autoimmune polyglandula syndrome type 1 (APS1). Genomics 35:322–326.

Yaspo ML, Rinderle C, Christensen H-M, Schweiger M, Blechschmidt K, Rosenthal A. 1998. Functional analysis of the AIRE gene associated with APECED and characterization of the murine AIRE homologue. Am J Hum Genet 63:A394.

Zuklys S, Balciunaite G, Agarwal A, Falser-Kan E, Palmer E, Hollander GA. 2000. Normal thymic architecture and negative selection are associated with Aire expression, the gene defective in the autoimmune polyendocrinopathy-candidiasis-extodermal dystrophy (APECED). J Immunol 165:1976–1983.

